# Fungi Dominated the Incorporation of ^13^C-CO_2_ into Microbial Biomass in Tomato Rhizosphere Soil under Different CO_2_ Concentrations

**DOI:** 10.3390/microorganisms9102121

**Published:** 2021-10-09

**Authors:** Hehua Wang, Juan Wang, Chaorong Ge, Huaiying Yao

**Affiliations:** 1 Research Center for Environmental Ecology and Engineering, School of Environmental Ecology and Biological Engineering, Wuhan Institute of Technology, Wuhan 430073, China; 15902711089@163.com; 2 Key Laboratory of Urban Environment and Health, Institute of Urban Environment, Chinese Academy of Sciences, Xiamen 361021, China; jwang@iue.ac.cn; 3 Ningbo Key Laboratory of Urban Environmental Processes and Pollution Control, Ningbo Urban Environment Observation and Research Station—NUEORS, Institute of Urban Environment, Chinese Academy of Sciences, Ningbo 315800, China

**Keywords:** eCO_2_, PLFA-SIP, fungi, actinomycete, microbial community structures

## Abstract

An elevated CO_2_ (eCO_2_) fumigation experiment was carried out to study the influence of various CO_2_ concentrations on microorganisms involved in the incorporation of root-derived C in greenhouse soil systems. In this study, 400 and 800 µmol·mol^−1^ CO_2_ fumigation treatments were conducted during tomato planting. Phospholipid fatty acid (PLFA) profiling based on the stable isotope probing (SIP) technique was applied to trace active microorganisms. The absolute total abundance of ^13^C-PLFAs was much higher under eCO_2_ treatment. Most of the ^13^C-CO_2_ was incorporated into the ^13^C-PLFAs 18:2ω6,9 (fungi), 16:0 (general PLFA), 18:1ω9c (Gram-negative bacteria, G^−^) and i17:0 (Gram-positive bacteria, G^+^) via rhizodeposition from tomato under ambient CO_2_ (aCO_2_) and eCO_2_ treatments, suggesting similar responses of active microorganisms to different CO_2_ treatments. However, the fungi (characterized by the ^13^C-PLFA 18:2ω6,9) played a much more dominant role in the incorporation of root-derived C under eCO_2_. Actinomycetes, marked by the ^13^C-PLFA 10-Me-18:0, occurred only on labeling day 15 under the eCO_2_ treatment, indicating that the actinomycetes fed on both soil organic carbon and fresh rhizodeposition. It was indicated that eCO_2_ significantly affected microbial biomass and microbial community structures involved in the incorporation of ^13^C-CO_2_ via tomato root secretions, as supported by Adonis analysis and the Mantel test.

## 1. Introduction

Atmospheric CO_2_ concentrations have been increasing annually since the Industrial Revolution, reaching 400 µmol·mol^−1^ for the first time in 2013 [1]. eCO_2_ could improve the net primary production and quality by accelerating photosynthetic rates and increasing water use efficiency [2,3,4,5]. At the same time, plant rhizodeposition, which accounted for 17% of photosynthates [6], was altered quantitatively and qualitatively under eCO_2_ treatment [4,7,8,9]. As a consequence, microbial biomass was generally significantly increased under eCO_2_ because of the increased carbon flow in plant root secretions [8,10], and the microbial community structure was affected by eCO_2_ to different degrees depending on plant species [11], soil characteristics [11] and experimental designs [12,13]. However, whether the changes in microbial biomass and community structure were derived from increased rhizodeposition or soil organic matter remains largely unknown in most cases [11,14,15], which could ultimately affect the soil’s nutrient balance and the sustainable development of ecosystems.

Under this condition, studies on the microorganisms responsible for the incorporation of root-derived C have received more and more attention and can provide insights into this phenomenon. Fungi (PLFAs 18:1ω9 and 18:2ω6,9) and G^−^ (PLFAs 16:1ω7, 18:1ω7 and cy19:0) incorporated most of the ^13^C-CO_2_ in a 5 h stable isotope labeling experiment in grassland soils [16]. Similarly, the ^13^C-PLFA 18:2ω6,9 was shown to significantly incorporate root-derived C, while bacterial PLFAs showed no significant changes in another experiment conducted on young beech trees [17]. For anaerobic incubation with ^13^C-labeled urea in wheat-rice rotation soils, the ^13^C-PLFA 18:1ω9c was much more abundant when the wheat season changed into the rice season [18]. Furthermore, microbes involved in the incorporation of root-derived C were found to be significantly different under various CO_2_ concentrations with the support of Deoxyribonucleic acid-stable isotope probing (DNA-SIP) [19]. Bacilli, Gammaproteobacteria and Clostridia were dominant under aCO_2_ treatment, while Bacilli and Betaproteobacteria were dominant under the eCO_2_ treatment. Based on the above discoveries, it was found that fungi (18:2ω6,9) played a dominant role in incorporating root-derived C, and the changes in active microbial community structures were mainly dependent on ecosystems. eCO_2_ stimulates the C flow from plant roots to soils and promotes microbial activity. Greater utilization of additional C resources and root secretions [20,21], a significantly higher abundance of genes associated with C and N cycling [22] and increased enzyme activity [23,24] indicate rapid nutrient cycling and the stimulation of microbial activity under eCO_2_. Unfortunately, the isotope pulse-labeling technique used in previous studies was less stable than the steady isotope labeling technique, and little research has focused on the effects of various CO_2_ concentrations, especially in greenhouse soil ecosystems.

Soils managed under plastic tunnel greenhouses, which are widely used in fruit and vegetable plantations and characterized by lower pH levels and nutrient-poor conditions under longer planting durations, have rarely been studied under various CO_2_ treatments [22,25,26]. The soil microbial community composition in the rhizosphere soils of tomato changed insignificantly under various CO_2_ treatments in our previous study [27]. However, the changes in active microbial community structures incorporating root-derived C were largely unknown. The Phospholipid fatty acid-stable isotope probing (PLFA-SIP) technique based on steady ^13^CO_2_ labeling can be used to reflect the changes in active PLFAs that are characteristic of diverse microbes [28].

A study demonstrated that the ideal concentration of CO_2_ for the production of vegetables in greenhouses is 800–1000 µmol·mol^−^^1^ [29]. It has been shown that eCO_2_ can significantly improve crop photosynthesis, including that in tomatoes [30,31]. According to reports by the Intergovernmental Panel on Climate Change (IPCC, 2014) [1], atmospheric CO_2_ has been reached 400 µmol·mol^−^^1^ in 2013, and it will increase at the rate of 1.9 μmol·mol^−^^1^·y^−^^1^, reaching 550 µmol·mol^−^^1^ in 2050. In previous studies about eCO_2_ fumigation, higher CO_2_ concentration was usually set as 550 [24], 700 [32] or 800 µmol·mol^−^^1^ [26,33,34] and so on, and the ambient atmospheric CO_2_ concentration was usually regarded as 400 µmol·mol^−^^1^. In order to clearly reveal the stimulation of plant biomass under eCO_2_ condition, 800 µmol·mol^−^^1^ CO_2_ was also chosen in our study. As higher plant dry biomass usually means more root secretions, so only one higher eCO_2_ concentration was chosen to induce significant changes of microbes.

In this study, a 2% ^13^C-CO_2_ steady-state labeling experiment with the support of the PLFA-SIP technique was conducted in both tomato planted and unplanted soils, in which 400 or 800 µmol·mol^−1^ CO_2_ fumigation was performed for 15 days because of the rapid metabolism of PLFAs. Based on our previous study that fungal biomass was significantly increased under eCO_2_, we hypothesized that fungi would play a dominant role in the incorporation of root-derived C under eCO_2_ treatment. The aim was to reveal the differences in the compositions of active microbial communities that assimilate plant-derived carbon under various CO_2_ concentrations.

## 2. Materials and Methods

### 2.1. Study Site

In August 2020, the plow layer of the soil was sampled from a 10-year-old tomato greenhouse located in Wuhan city (30°17’44″ N, 114°16’34″ E). The field holding capacity of the soil was 24.36%, and the moisture content of the air-dried soil was 2.86%. The soil pH was 5.31 (soil:water = 1:2.5). The total carbon and total nitrogen contents were 10.3 and 1.6 g·kg^−1^ dry soil, respectively, and the C:N ratio was 6.49. The dissolved organic carbon (DOC) and dissolved organic nitrogen (DON) contents were 395.75 and 122.88 mg·kg^−1^ dry soil, respectively. The soil NH_4_^+^-N and NO_3_^−^-N contents were 2.11 and 37.16 mg·kg^−1^ dry soil, respectively. The soil was defined as loamy because sand, silt and clay accounted for 47.7%, 42.6% and 8.2% of its contents, respectively.

### 2.2. Experimental Set-Up

In this study, each pot was filled with the equivalent of 200 g of soil (dry weight). The soils in each pot were passed through a 5.0 mm sieve, the water content was adjusted to 65% of the field holding capacity, and 0.2 g NPK fertilizer was mixed before the transplantation of the tomato plants.

The seeds of the tomato plants (*Solanum lycopersicum* L., Jinguan 28) were soaked in tap water (37 °C, 10 h) and then germinated at room temperature (25 °C) according to the planting instructions. Tomato seedlings were placed into larger pots (15 × 12 × 14 cm) for seedling cultivation. After 13 days, three plants of similar sizes (5 cm length with 4 leaves) were transplanted into each small pot (9 × 6 × 9.5 cm). All the pots were transferred to a controlled environment room (day 22–26 °C/night 15–19 °C; photoperiod, 12 h light; watered every two days with deionized water; CO_2_ concentration, 400 µmol·mol^−1^, controlled with a volumetric flow meter and a control panel).

All pots were divided into two parts: pots for plant growth and pots for soil incubation. In each part, two variants (CO_2_ concentration and labeling days) were set. In total, 28 pots for plant growth (2 CO_2_ concentrations (400 and 800 µmol·mol^−1^ CO_2_) × 3 labeling days (labeling days 5, 10 and 15) × 4 replicates + 4 pots sampled on the day before labeling); 12 pots for soil incubation (2 CO_2_ concentrations (400 and 800 µmol·mol^−1^ CO_2_) × 1 labeling days (labeling days 15) × 4 replicates + 4 pots sampled on the day before labeling).

One week after transplantation (day 21), the pots were separated equally into two different CO_2_ concentration treatment groups (400 or 800 µmol·mol^−1^ CO_2_), and 2% ^13^C-CO_2_ steady-state labeling was performed. The two growth chambers were placed in the above-mentioned controlled-environment room, so the growth conditions were the same as those in the plant growth period. The steady-state labeling of ^13^CO_2_ was accomplished by mixing compressed air without CO_2_, natural ^12^CO_2_ and ^13^CO_2_ at fixed rates controlled by mass-flow controllers, which were described previously [30]. Briefly, the flow rates of ^13^CO_2_, ^12^CO_2_ and air without CO_2_ were set as 0.072 mL·min^−1^, 7.128 mL·min^−1^ and 18 L·min^−1^, respectively, in the 400 µmol·mol^−1^ CO_2_ chamber, while they were 0.144 mL·min^−1^, 14.256 mL·min^−1^ and 18 L·min^−1^, respectively, in the 800 µmol·mol^−1^ CO_2_ chamber.

In the pots used for plant growth, destructive sampling of tomato plants and rhizosphere soils was performed on days 21 (the day before labeling), 26, 31 and 36 (every 5 days after labeling). From the pots used for soil cultivation, soils were collected only on day 21 (the day before labeling) and day 36 (the end of the labeling experiment). Rhizosphere soils were harvested by collecting the soils adhering to the root after shaking and then freeze-dried for PLFA analysis. Plant shoots and roots were separated and dried (105 °C for 45 min and 60 °C for 48 h) for further analysis.

### 2.3. Analysis of Biochemical Properties

To reflect the changes in the biochemical properties of the soils, the soil pH, moisture, DOC, DON, NO_3_^−^-N and NH_4_^+^-N were measured. The measurement details are available in a previous study [27]. In brief, the total C and total N levels in the soils were measured by an elemental analyzer (Elementar Vario MACRO cube, Langenselbold, Germany) according to the operating instructions. The NO_3_^−^-N and NH_4_^+^-N in the soil were extracted with 2 M KCl at a 1:5 fresh soil to KCl ratio, and the extract was analyzed with a continuous flow analyzer (SKALAR, Delft, The Netherlands). The DOC and DON in the soil were extracted with 0.5 M K_2_SO_4_ at a 1:5 fresh soil to K_2_SO_4_ ratio and analyzed with a Multi N/C 2100S TOC/TN b analyzer (Analytik Jena, Jena, Germany). The % C and 13C/12C ratio of the soils and tomato roots and shoots were analyzed with a Flash EA 2000 Series Elemental Analyzer connected via a Conflo IV to a Delta V Advantage isotope ratio mass spectrometer (all Thermo Scientific, Germany) [35]. The isotope ratios were calculated according to a previous study [36].

### 2.4. PLFA Analysis

PLFA extraction and analyses were performed according to the modified Bligh and Dyer method [37], the details of which were provided in a previous study [27]. The concentration and ^13^C labeling of fatty acid methyl esters (FAMEs) were analyzed by using a Trace GC 1310 system with a combustion column attached via a GC IsoLink II system to a Delta V Advantage isotope ratio mass spectrometer (all Thermo Scientific, Germany) [36]. Other details were the same as those described in previous studies [36,38].

In total, 38 PLFAs were detected across all the treatments. Taking into account the concentration of ^13^C among these 38 PLFAs, 19 PLFAs accounting for 90% of the total amount of PLFAs were considered valid and were finally retained for further analyses. These PLFAs were divided into the following categories according to previous reports [39]: general PLFAs (14:0, 15:0, 16:0, 17:0, 18:0 and 20:0), PLFAs from G^-^ (16:1ω7c, cy17:0, 18:1ω9c and cy19:0), PLFAs from G^+^ (i15:0, a15:0, i16:0, i17:0 and a17:0), actinomycetes (10-Me-16:0 and 10-Me-18:0), fungi (18:2ω6,9) and arbuscular mycorrhizal fungi (AMF) (16:1ω5c) [40].

The proportion (Pi) of ^13^C in each PLFA was determined according to the mass balance equation below [36].

P_i_ = (AT% ^13^C_t_ − AT% ^13^C_0_)/(AT% ^13^C_g_ − AT% ^13^C_0_) (1)

where AT% means the ratio of an isotope atom to the total atomic number of an element, AT% ^13^C_t_ and AT% ^13^C_0_ are the AT% ^13^C/^12^C enrichment (%) of each PLFA in the rhizosphere soil at the end and beginning of labeling, respectively, and AT% ^13^C_g_ is the AT% ^13^C/^12^C enrichment (%) of labeled CO_2_ (%). The absolute amount of labeled ^13^C in each PLFA was defined as the product of Pi and the absolute concentration of carbon in each PLFA (the number of carbons multiplied by the absolute abundance of each PLFA).

### 2.5. Statistical Analysis

Basic data processing, including the calculation of means and standard deviations (SDs), was performed with Microsoft Excel 2010. Tests of significant variation in the effects of CO_2_ concentrations and labeling days on biochemical properties and plant biomass were conducted with the Scheirer–Ray–Hare test in R 3.6.3 with the rcompanion package [41]. Analyses of the effects of CO_2_ concentrations and labeling days on microbial community structure (PLFAs) were conducted via Adonis analysis with 999 permutations in R 3.6.3. The assessment of whether the mol% of PLFAs or ^13^C was correlated with environmental factors was carried out with Mantel tests. The principal component analysis (PCA) of 19 PLFAs was conducted with the help of IBM SPSS Statistics 22. Other figures were plotted by using Origin 2018.

## 3. Results

### 3.1. Effect of CO_2_ Concentration on the Tomato Biomass and AT% ^13^C/^12^C

**Figure 1:**
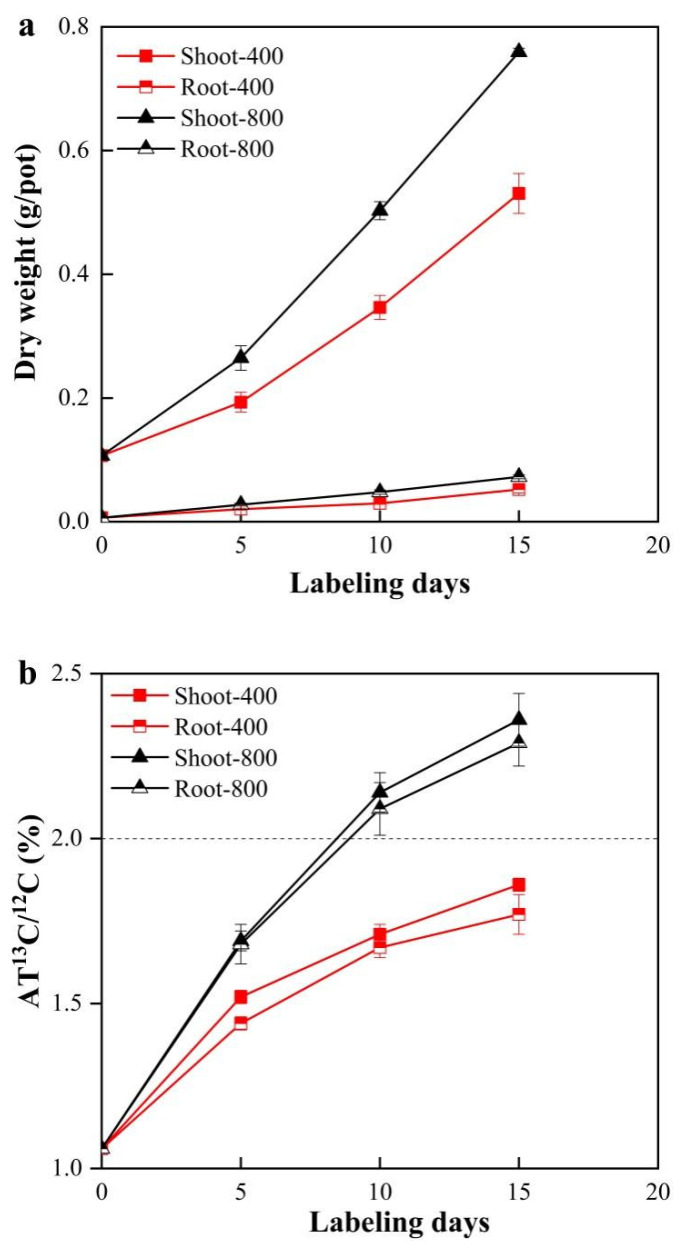
Changes in plant dry weights (**a**) and AT% ^13^C/^12^C values of shoots and roots (**b**) during the 15 labeling days. 400:400 µmol·mol^−1^ CO_2_ treatment; 800:800 µmol·mol^−1^ CO_2_ treatment. Bars represent the standard errors of the means (n = 4).

The eCO_2_ concentration significantly (*p* < 0.001) increased the shoot and root dry weights of tomato at the end of the labeling experiment (Figure 1a). However, both the labeling days and CO_2_ concentration:labeling days had no effect on plant biomass during the whole labeling period (*p* > 0.05).

The natural AT% ^13^C/^12^C values of the tomato shoots and roots were 1.06 before labeling. The AT% ^13^C/^12^C values of the shoots and roots were significantly (*p* < 0.001) higher under the eCO_2_ treatment, while the CO_2_ concentration:labeling days produced no significant (*p* > 0.05) effects on these induces during the labeling period (Figure 1b). Specifically, the AT% ^13^C/^12^C values of both tomato shoots and roots increased significantly (*p* < 0.05) with the labeling days under both the 400 and 800 µmol·mol^−1^ CO_2_ treatments. Moreover, the AT% ^13^C/^12^C values of the shoots and roots were greater than 2 (the AT% ^13^C/^12^C value of CO_2_ used in this experiment) on labeling days 10 and 15 under the 800 µmol·mol^−1^ CO_2_ treatment (Figure 1b), indicating that most of the carbon in the tomato shoots and roots was derived from photosynthesate after labeling day 10 under eCO_2_.

### 3.2. Effect of CO_2_ Concentration on the Biochemical Properties of the Soils

**Figure 2:**
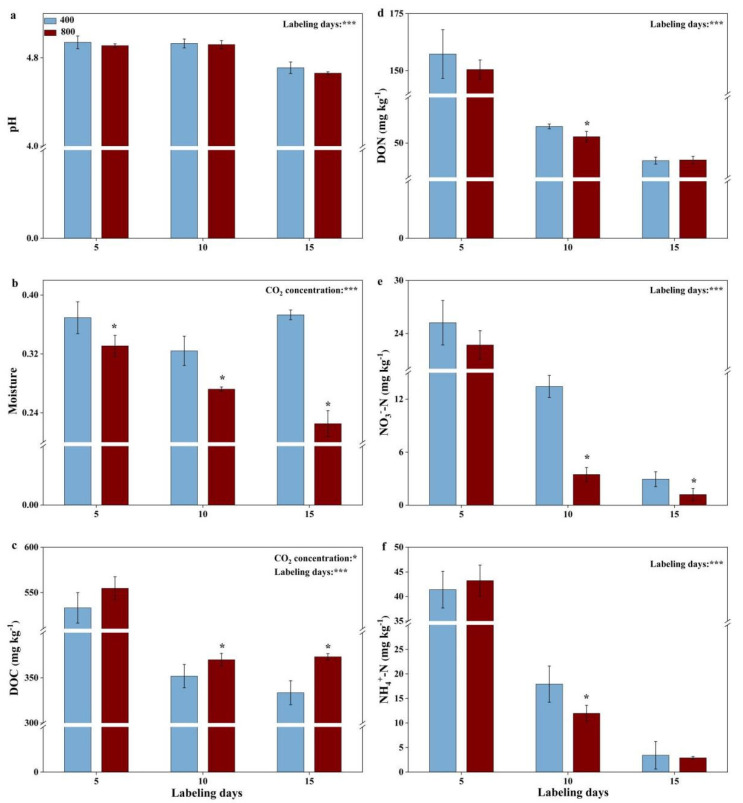
Changes in pH (**a**), moisture (**b**), DOC (**c**), DON (**d**), NO_3_^−^−N (**e**) and NH_4_^+^−N (**f**) during the 15 labeling days under both the 400 and 800 treatments. 400:400 µmol·mol^−1^ CO_2_ treatment; 800:800 µmol·mol^−1^ CO_2_ treatment. Bars represent the standard errors of the means (*n* = 4). *: *p* < 0.05; ***: *p* < 0.001.

The changes in the biochemical properties of the soils during the labeling period were analyzed. In the planted treatment groups, the soil pH was nonsignificantly influenced (*p* > 0.05) by the CO_2_ concentration but decreased significantly (*p* < 0.001) with labeling days (Figure 2a). The soil moisture decreased significantly (*p* < 0.05) under the eCO_2_ treatment (Figure 2b). The CO_2_ concentration significantly (*p* < 0.05) increased the soil DOC, while labeling days significantly (*p* < 0.05) decreased it, and CO_2_ concentration:labeling days had no effect (*p* > 0.05) during the whole labeling period (Figure 2c). In particular, the soil DOC increased significantly (*p* < 0.05) with the CO_2_ concentration but decreased significantly (*p* < 0.001) with labeling days. The soil DON was not influenced by the CO_2_ concentration (*p* > 0.05) but decreased significantly (*p* < 0.001) with labeling days over the entire incubation period (Figure 2d). Similar to the soil DON, both the soil NO_3_^−^-N and NH_4_^+^-N decreased significantly (*p* < 0.001) with labeling days and were much lower (*p* < 0.05) under the eCO_2_ treatments on labeling days 10 and 15 (Figure 2e,f). In the soil cultivation treatments, the soil DOC increased significantly (*p* < 0.05), while the soil NO_3_^−^-N and soil NH_4_^+^-N decreased significantly (*p* < 0.05), under the eCO_2_ treatment (Table S1). The AT% ^13^C/^12^C values of the soils remained at 1.082 before labeling day 5 in both the aCO_2_ and eCO_2_ treatments, while they increased significantly (*p* < 0.05) on labeling days 10 and 15 under the eCO_2_ treatment.

### 3.3. Effect of CO_2_ Concentration on the Soil Microbial Community

In the tomato-planted treatment groups, the absolute abundance of total PLFAs was significantly (*p* < 0.05) higher under the eCO_2_ treatments (30.8 ± 0.6 vs. 38.5 ± 3.6 nmol·g^−1^ soil) at the end of labeling. However, the total PLFA contents of soils from the unplanted treatment groups were not significantly (*p* > 0.05) affected by the CO_2_ concentration, as the values in the 400 and 800 µmol·mol^−1^ CO_2_ treatment groups were 19.3 ± 0.4 and 20.1 ± 0.2 nmol·g^−1^ soil, respectively.

Based on the distributions of 19 PLFAs on each sampling day, general bacteria (PLFA 16:0), G^−^ (PLFAs cy19:0 and 18:1ω9c) and fungi (PLFA 18:2ω6,9c) were considered to be most abundant in both the planted and unplanted treatment groups (Figure S1). However, minor differences in the relative abundance of the abovementioned PLFAs existed between the planted and unplanted treatment groups. For example, the abundance of the fungal PLFA marker 18:2ω6,9 was much higher (*p* < 0.05) in the planted treatment groups than in the unplanted treatment group. For the significantly (*p* < 0.05) changed microbial PLFAs under various CO_2_ concentrations, both the PLFA species and numbers (6, 3 and 5) changed with labeling days in the planted treatment groups. Specifically, the relative abundance of the PLFA 18:2ω6,9 (fungi) was not significantly affected by eCO_2_ in the earlier labeling period (day 5) but was affected by eCO_2_ on labeling days 10 and 15, finally accounting for more than 20% of the total PLFAs at the end of labeling (Figure S1).

**Figure 3:**
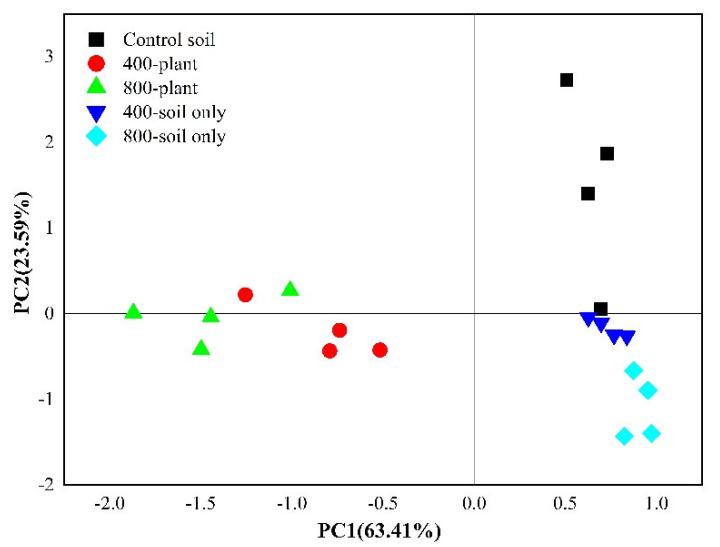
Principal component analysis of the PLFA composition of soil samples at the end of the 15−day labeling experiment. Values in parentheses on the axis labels indicate the percentage variation accounted for by each axis. 400:400 µmol·mol^−1^ CO_2_ treatment; 800:800 µmol·mol^−1^ CO_2_ treatment; the control soil is the original tomato soil.

Principal component analysis (PCA) based on the relative abundance of 19 PLFAs was conducted to reveal the effects of tomato plants and CO_2_ concentrations on the microbial community composition (Figure 3). The microbial community compositions in the planted and soil cultivation treatment groups were separated on the first principal component axis (PC1), explaining 63.41% of the total variation, indicating the important role of tomato plants in shaping the microbial community structure. Analyses of the loading scores of PLFAs on the PC1 axis suggested that significant (*p* < 0.05) increases in i16:0 (score 0.081) and 10-Me-16:0 (score 0.081) and decreases in 18:2ω6,9 (score −0.082) and i17:0 (score −0.068) occurred in the soils without tomato (Figure S2), which was the same as the distribution of the mol% of PLFAs (Figure S1). Though the microbial community structures of soils from the 400-plant and 800-plant treatment groups were nicely clustered by direct visual observation (Figure 3), the *p* value of the Adonis analysis was above 0.05, which indicated that the CO_2_ concentration had no significant effects on microbial community structure in soils from the planted treatments during the 15 days of CO_2_ fumigation.

### 3.4. Variation in Soil Microbial Composition Incorporating ^13^C-Rhizodeposition under Different CO_2_ Concentrations

In the tomato planted treatment groups, the total ^13^C incorporation into PLFAs was significantly stimulated by the CO_2_ concentration (*p* < 0.05) and labeling days (*p* < 0.001), reaching 188.71 ± 16.14 and 456.76 ± 67.91 nmol·g^−1^ soil in the 400-day 15 and 800-day 15 treatment groups, respectively (Table 1). The average enrichment rate of ^13^C under the eCO_2_ treatment was almost twice as high as that under the aCO_2_ treatment (69% vs. 36%) (Table 1). However, in the soil incubation treatment groups, the average enrichment rate of ^13^C was less than 2%, and ^13^C labeling was not affected by the CO_2_ concentration (*p* > 0.05) (Table 1). Based on the distribution of ^13^C among fungal, general, G^−^, G^+^ and actinomycete PLFAs, fungi and general bacteria were dominant in the planted treatments, while general bacteria and G^−^ were dominant in the unplanted treatments (Figure S3).

**Figure 4:**
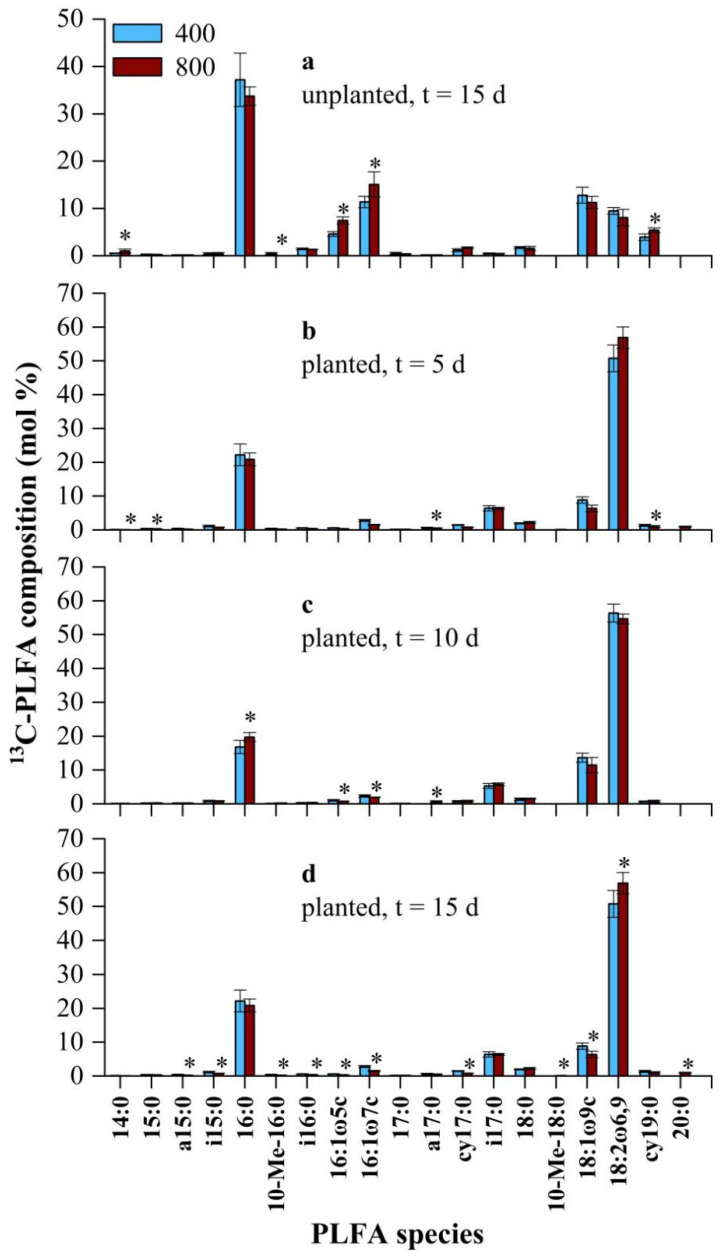
Differences in ^13^**C**-PLFA composition (mol%) between the 400 and 800 µmol·mol^−1^ CO_2_ treatment groups in unplanted (**a**) and planted (**b**–**d**) soils on each sampling day. Labels shown in the figure represent significant ^13^C-PLFA biomarkers (*p* < 0.05). 400:400 µmol mol^−1^ CO_2_ treatment; 800:800 µmol·mol^−1^ CO_2_ treatment. Bars represent the standard errors of the means (*n* = 4). *: *p* < 0.05.

The percentage distribution of ^13^C in the PLFAs revealed the composition of microorganisms that incorporated root-derived ^13^C (Figure 4). In the unplanted soils, the ^13^C in a general PLFA (16:0) and G^−^ (16:1ω7c and 18:1ω9c) accounted for 60% of the total ^13^C (Figure 4a), which was in accordance with previous results (Figure S3). In contrast, 18:2ω6,9 (fungi), 16:0 (general PLFA), 18:1ω9c (G^−^) and i17:0 (G^+^) were the most abundant ^13^C-PLFAs under both 400 and 800 µmol·mol^−1^ CO_2_ in the planted treatment groups, accounting for more than 85% of the total ^13^C-PLFAs (Figure 4b–d).

**Figure 5:**
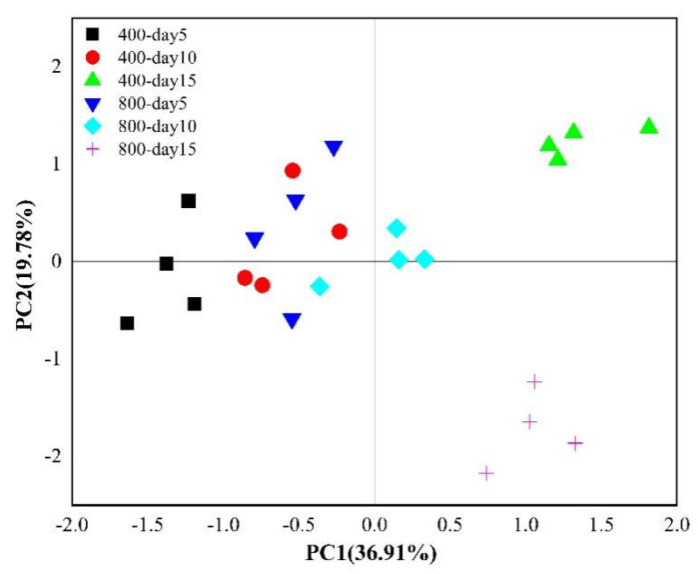
Principal component analysis of the ^13^C−PLFA composition (mol%) of soil samples under various CO_2_ treatments during the 15-day labeling experiment. Values in parentheses on the axis labels indicate the percentage variation accounted for by each axis. 400:400 µmol·mol^−1^ CO_2_ treatment; 800:800 µmol·mol^−1^ CO_2_ treatment.

The differences in the soil microbial community structure in terms of ^13^C incorporation between the 400 and 800 µmol·mol^−1^ CO_2_ treatment groups was dependent on the labeling days, as indicated by both PCA and Adonis analyses (Figure 5, Table S2). Adonis analyses demonstrated that significant (*p* < 0.05) differences in the microbial community structure between the 400 and 800 µmol·mol^−1^ CO_2_ treatment groups occurred on labeling days 10 and 15 (Table S2). PCA further revealed that the 400-day 10 and 800-day 10 treatment groups were separated by PC1, while the 400-day 15 and 800-day 15 treatment groups were separated by PC2. In detail, the plant-derived ^13^C contents of the PLFAs 16:0 (a general PLFA), 18:1ω9c (G^−^) and 16:1ω7c (G^−^) were much higher (*p* < 0.05) under the 400 µmol·mol^−1^ CO_2_ treatment, while the plant-derived ^13^C contents of the PLFAs 18:2ω6,9 (fungi), 20:0 (general PLFA) and 10-Me-18:0 (actinomycetes) were much higher (*p* < 0.05) under the 800 µmol mol^−1^ CO_2_ treatment at the end of labeling period (Figure 4d). In terms of the AT% ^13^C/^12^C values of PLFAs, 18:2ω6,9 and 18:1ω9c were the top two of the 19 discussed ^13^C-PLFAs (Figure S4). Moreover, the AT% ^13^C/^12^C values of 18:2ω6,9 exceeded 2 (the AT% ^13^C/^12^C value of CO_2_ used in this experiment) on labeling days 10 and 15 under in 800 µmol·mol^−1^ CO_2_ treatment group (Figure S4). In addition, the incorporation of ^13^C from CO_2_ into the PLFAs 10-Me-18:0 and 20:0 occurred only in the 800-day 15 treatment group (Figure 4b–d), although the difference in the mol% of the PLFAs 10-Me-18:0 and 20:0 between the 400-day 15 and 800-day 15 treatment groups was not significant (*p* > 0.05), suggesting that microorganisms containing the PLFAs 10-Me-18:0 and 20:0 were dependent mainly on the original soil carbon source (such as organic matter) in the 400 µmol·mol^−1^ CO_2_ treatment group and in the earlier period of eCO_2_ treatment (up to labeling day 10).

### 3.5. Relationship between PLFAs and Environmental Factors

To reveal the associations between microbial compositions and soil and plant characteristics, Mantel test analyses were conducted based on the mol% of PLFAs or ^13^C and three key factors (CO_2_ concentration, DOC and total dry weight (TDW)). The whole PLFA profile was significantly (*p* < 0.05) related to DOC and TDW, while the whole ^13^C-PLFA profile was significantly (*p* < 0.05) correlated with CO_2_, DOC and TDW (Table 2). However, when TDW or DOC was set as a covariate, the influence of DOC or TDW on the whole PLFA profile was not significant (*p* > 0.05), indicating that the soil DOC and plant TDW interacted with each other. When TDW or DOC was set as a covariate, the effect of CO_2_ on the whole ^13^C-PLFA profile was not significant (*p* > 0.05), indicating that CO_2_ made an influence on ^13^C-PLFA profile by stimulating the TDW and DOC. Furthermore, when DOC was set as a covariate, the effect of TDW on the whole ^13^C-PLFA profile was significant (*p* < 0.05), suggesting that TDW could directly influence microbial community structures.

When specific PLFAs were considered alone, the results showed that CO_2_ produced a significant (*p* > 0.05) influence on ^13^C-18:1ω9c by increasing the tomato biomass, while it had a direct influence on 18:1ω9c (*p* < 0.05) (Table S3). In addition, both 18:2ω6,9 and ^13^C-18:2ω6,9 were significantly (*p* < 0.05) related to TDW. However, CO_2_ and TDW were significantly (*p* < 0.05) correlated with only the labeled ^13^C-10-Me-18:0 and ^13^C-20:0 (Table S3). The above results indicated that specific ^13^C- PLFAs were correlated with environmental factors in various manners.

## 4. Discussion

Similar responses of soil microorganisms to root-derived C under aCO_2_ have been shown in previous studies [16,17]. The ^13^C-PLFA 18:2ω6,9 was shown to significantly (*p* < 0.05) incorporate root-derived C, while bacterial PLFAs showed no significant changes in an experiment conducted on young beech trees [17]. In our results, the significantly higher abundance of ^13^C among fungal and actinomycete PLFAs under eCO_2_ treatment partially supports our hypothesis and is consistent with previous findings.

### 4.1. Effect of CO_2_ Concentration on ^13^C-Plant Biomass

The AT% ^13^C/^12^C values of tomato shoots and roots were significantly (*p* < 0.05) increased in the 800 µmol·mol^−1^ CO_2_ treatment group (Figure 1b), in accordance with the general theory that eCO_2_ is beneficial for plant photosynthesis [30,31]. In addition, the greater tomato root biomass and AT% ^13^C/^12^C values of tomato roots (exceeding 2, the AT% ^13^C/^12^C of CO_2_ value used in this experiment) (Figure 1) might indicate that the quantity of ^13^C-root secretions increased in the 800 µmol·mol^−1^ CO_2_ treatment group, as rhizodeposition accounted for approximately 17% of the photosynthates [6] and an increase in fine roots usually indicates increased root secretion. The significant (*p* < 0.05) increases in soil DOC (Figure 2c) and ^13^C among the PLFAs (Table 1) observed under eCO_2_ further supported the above hypothesis.

### 4.2. Effect of CO_2_ Concentration on Microbial Community Structure

During the 15 days of CO_2_ fumigation, the absolute abundance of the total PLFAs was significantly (*p* < 0.05) stimulated by eCO_2_ treatment, while the overall microbial community structure (the PLFA profile) was not influenced by CO_2_ concentration (Figure 3), as confirmed by Adonis analysis and Mantel tests (*p* > 0.05). The above results were similar to those of previous studies in which short-term eCO_2_ fumigation usually led to increases in root rhizodeposition and microbial biomass [42] but had little influence on microbial community structure [27,43,44]. In fact, because of the differences in plant species, soil characteristics and experimental designs, the observed response of the microbial community structure to eCO_2_ is not always the same [27]. A study conducted in a grassland revealed that even a longer period of eCO_2_ fumigation produced no effect on the microbial community structure [45], which might be attributed to the specific ecosystem involved and the large pool of soil carbon underground. Therefore, a focus on the changes in microbes involved in the incorporation of root-derived C is essential for understanding the effect of eCO_2_.

### 4.3. Effect of CO_2_ Concentration on ^13^C-Microbial Community Structure

In this study, the ^13^C-PLFAs 18:2ω6,9, 16:0, 18:1ω9c and i17:0 showed the highest activity in terms of the incorporation of rhizodeposited ^13^C compared with other PLFAs under both 400 and 800 µmol·mol^−1^ CO_2_, accounting for 85% of the total ^13^C (Figure 4d), which was in accordance with previous studies conducted in forests or other nonflooded ecosystems [16,17]. Moreover, both the absolute abundance of total ^13^C (Table 1) and the distribution of ^13^C-PLFAs (Figure 5) changed significantly (*p* < 0.05) under eCO_2_ at the end of the labeling period.

On the one hand, ^13^C-18:2ω6,9 (fungi) showed a significantly (*p* < 0.05) higher relative abundance under eCO_2_ (56.9% vs. 50.8%) (Figure 4d). The dominant role of fungi (18:2ω6,9) in incorporating root-derived carbon has also been observed in previous studies [16,17,46]. The significantly increased relative abundance of the ^13^C-PLFA 18:2ω6,9 under eCO_2_, in accordance with experiments conducted in young beech trees [17], could be attributed to the characteristics of fungi. First, the suitable pH range for fungi is acidic; in the present study, it was 4.8 on average and decreased under eCO_2_ (Figure 2a) [47]. Second, fungi can efficiently absorb nutrients through their long hyphal network to overcome the significant decrease in the soil N status, especially in the later stages of eCO_2_ fumigation (Figure 2d–f) [48]. Third, mutualism occurs between AMF and plant roots, and eCO_2_ further stimulates the growth of AMF and plants by increasing plant rhizodeposition secretion into rhizosphere soils [49,50]. Finally, fungi contain phenol oxidase, and this enzyme can mediate the absorption of sufficient N resources from recalcitrant C forms (such as lignin) with increased levels under eCO_2_ [51,52]. Therefore, the increase in fungi might be a general trend for eCO_2_ treatments in most studies. In addition, Mantel tests suggested that ^13^C-18:2ω6,9 was significantly (*p* < 0.05) related to TDW (Table S3), which means that fungi were affected mainly by the plant biomass or root secretions in this study. Therefore, the above results were consistent with the hypothesis that fungi played a much more dominant role in the incorporation of root-derived carbon under the eCO_2_ treatment.

On the other hand, 20:0 (general PLFA) and 10-Me-18:0 (actinomycete) were involved in the incorporation of ^13^C-CO_2_ later in the eCO_2_ treatment (such as 15 days) (Figure 4d). However, no significant (*p* > 0.05) variations in the mol% of 20:0 or 10-Me-18:0 existed between the 400 and 800 µmol·mol^−1^ CO_2_ treatment groups (Figure S1d). This result suggested that C among these two PLFAs was mainly derived from original soil carbon sources under aCO_2_ treatment, but root-derived carbon (rhizodeposition) could also be utilized under the eCO_2_ treatment, which was in accordance with the wide range of substrates utilized by actinomycetes [53]. Mantel tests demonstrated that ^13^C-20:0 and ^13^C-10-Me-18:0 were significantly (*p* < 0.05) correlated with the CO_2_ concentration, TDW and CO_2_ _TDW (Table S3). Therefore, both general bacteria and actinomycetes also play an important role in the incorporation of root-derived carbon under long-term eCO_2_ fumigation [19].

The PLFA 18:1ω9c is usually regarded as a biomarker of G^−^ and fungi in agroecosystems and forest ecosystems, respectively [40]. In this study, ^13^C-18:1ω9c (G^−^) was much more abundant (*p* < 0.05) under aCO_2_ than under eCO_2_ (8.8% vs. 6.3%) on labeling day 15 (Figure 4d). The Mantel test results further indicated that the CO_2_ concentration affected ^13^C-18:1ω9c by increasing the TDW (Table S3). A recent study of the anammox reaction of urea revealed that the relative abundance of ^13^C-PLFA 18:1ω9c increased significantly (*p* < 0.05) during crop rotation from wheat to rice [18]. This might explain why ^13^C-18:1ω9c was much more abundant under aCO_2_, and it could be attributed to the higher water status under aCO_2_ (Figure 2b).

In addition, the whole ^13^C-PLFA profile was also identified to be significantly related to CO_2_ concentration (Mantel test, *p* < 0.05) (Table 2). The increase in plant biomass was considered to be the main mechanism underlying the above responses.

## 5. Conclusions

In this study, we focused on the changes in the overall microbial community structure and the subset of microbes involved in ^13^C-CO_2_ incorporation during 15 days of CO_2_ fumigation at different levels. We demonstrated that the overall microbial community structure was not changed, while the microbes involved in ^13^C-CO_2_ incorporation significantly (*p* < 0.05) differed between the 400 and 800 µmol·mol^−1^ CO_2_ treatment groups from labeling day 10 onward. The Mantel test further revealed that the ^13^C-PLFA profile was significantly related to the CO_2_ concentration. In addition, the mol% of ^13^C in the PLFAs 18:2ω6,9 (fungi), 20:0 (general PLFA) and 10-Me-18:0 (actinomycetes) were higher (*p* < 0.05) in the 800 µmol mol^−1^ CO_2_ treatment group, while those of 18:1ω9c (G^−^) and 16:1ω7c (G^−^) were much higher (*p* < 0.05) in the 400 µmol·mol^−1^ CO_2_ treatment group. In summary, this study successfully revealed the important role of 18:2ω6,9, 10-Me-18:0 and 18:1ω9c in incorporating rhizodeposits under eCO_2_. The structures of ^13^C rhizodeposits need to be analyzed to completely understand the mechanism of the effects of eCO_2_ on soil microbes in future work.

## Figures and Tables

**Table 1:** The total enrichment of ^13^C into the PLFA of individual soil samples under varied CO_2_ concentration treatments.

Labeled Days	CO_2_ Concentration (µmol·mol^−1^)	Labeled Amount (nmol·g ^−1^) Average ± STD	Enrichment Rate (%) Average ± STD
5	400 ^1^	24.43 ± 4.39 ab ^2^	7.43 ± 1.35 b
	800	60.05 ± 11.37 b	15.13 ± 1.45 c
10	400	49.66 ± 9.6 ab	12.65 ± 1.95 c
	800	150.46 ± 45.25 c	34.27 ± 6.29 d
15	400	188.71 ± 16.14 c	35.82 ± 2.33 d
	800	456.76 ± 67.91 d	68.89 ± 4.25 e
CO_2_ concentrations	*		
Labeling days	***		
CO_2_ concentration:Labeling days	ns		
Unplanted soil	400	3.52 ± 0.45 a	1.08 ± 0.14 a
	800	5.61 ± 0.79 a	1.65 ± 0.22 a

^1^ 400:400 µmol·mol^−1^ CO_2_ treatments; 800:800 µmol·mol^−1^ CO_2_ treatments. ^2^ Values are means ± standard deviation (n = 4). Values with different lowercase letters within a column and same stage are statistically significantly different at *p* < 0.05. * *p* < 0.05; *** *p* < 0.001.

**Table 2:** Mantel tests between PLFA or ^13^C-PLFA and environmental factors under 400 and 800 µmol·mol^−1^ CO_2_ treatments.

Factors	PLFA Composition		^13^C-PLFA Composition	
	Mantel Statistic r	Significance	Mantel Statistic r	Significance
CO_2_	0.3887	0.0562	0.4556	0.0289 *
DOC ^1^	0.3636	0.0489 *	0.4281	0.0256 *
TDW	0.4905	0.0204 *	0.7088	0.0008 ***
CO_2__DOC	0.1648	0.1881	0.1958	0.1594
CO_2__TDW	−0.0714	0.6301	−0.4199	0.9494
DOC_TDW	−0.02641	0.5555	−0.2638	0.846
TDW_DOC	0.3543	0.0685	0.6581	0.0035 **

^1^ Abbreviations: DOC, dissolved organic carbon; TDW, total dry weight; CO_2__DOC, the DOC was set as a covariate; CO_2__TDW, the TDW was set as a covariate; DOC_TDW, the TDW was set as a covariate; TDW_DOC, the DOC was set as a covariate. *, *p* < 0.05; **, *p* < 0.01; ***, *p* < 0.001.

## Data Availability

The data presented in this study are available in this article and Supplementary Materials.

## Supplementary Materials

The following are available online at https://www.mdpi.com/article/10.3390/microorganisms9102121/s1. Figure S1: Differences in PLFA composition (mol%) between the 400 and 800 µmol·mol^−1^ CO_2_ treatment groups in unplanted and planted soils on each sampling day. Figure S2: Loading scores of 19 PLFAs in the principal component analysis (PCA) at the end of the 15-day labeling experiment. Figure S3: ^13^C-PLFA compositions (mol%) of fungi, general PLFAs, G^−^, G^+^ and actinomycetes in planted and unplanted soils during the 15-day labeling experiment. Figure S4: Changes in AT% ^13^C/^12^C values of 18:2ω6,9 and 18:1ω9c under various CO_2_ treatments during the 15-day labeling experiment. Table S1: Biochemical properties of the unplanted soils under various CO_2_ treatments. Table S2: Adonis analysis based on mol% of ^13^C-PLFAs between the 400 and 800 µmol·mol^−1^ CO_2_ treatment groups during the whole labeling period. Table S3: Mantel tests between special PLFAs or ^13^C-PLFAs and environmental factors at the end of labeling in the 400 and 800 µmol·mol^−1^ CO_2_ treatment groups (*p*).
