# Supplementary material for: Fungi Dominated the Incorporation of 13C-CO2 into Microbial Biomass in Tomato Rhizosphere Soil under Different CO2 Concentrations
Source: Microorganisms. 2021 Oct 9;9(10):2121. doi: 10.3390/microorganisms9102121 (PMC8537487; doi:10.3390/microorganisms9102121)
Supplement: Supplementary file 1 [file microorganisms-09-02121-s001.zip › microorganisms-1372556-supplementary.pdf]

Table S1. Biochemical properties of the soils under varied CO_2 concentration in the unplanted treatments.

**CO_2 concentration (μmol·mol^{-1})**	**400[1]**	**800**
pH	4.43 ± 0.03 a[3]	4.48 ± 0.04 a
moisture	0.35 ± 0 a	0.36 ± 0.01 a
DOC[2] (mg kg^{-1})	351.9 ± 24.49 a	380 ± 10.23 b
DON (mg kg^{-1})	103.6 ± 4.73 a	113.3 ± 13.07 a
NO_3^--N (mg kg^{-1})	71.52 ± 1.73 b	66.62 ± 2.38 a
NH_4^+-N (mg kg^{-1})	16.06 ± 2.64 b	12.85 ± 1.3 a

[1] 400: 400 μmol·mol^{-1} CO_2 treatments; 800: 800 μmol·mol^{-1} CO_2 treatments.

[2] Abbreviations: DOC, dissolved organic carbon; DON, dissolved organic nitrogen.

[3] Values are means ± standard deviation (n = 4). Values with different lowercase letters within a column and same stage are statistically significantly different at $P < 0.05$.

Table S2. Adonis analysis based on mol% of ^{13}C-PLFA between 400 and 800 μmol·mol^{-1} CO_2 treatments during the whole labeling period.

	400-day5[1]	800-day5	400-day10	800-day10	400-day15	800-day15
400-day5						*
800-day5						*
400-day10				*	*	*
800-day10			*		*	*
400-day15			*	*		*
800-day15	*	*	*	*	*	

[1]Abbreviations: 400-day5, 400 μmol·mol^{-1} CO_2 treated for 5 days; 800-day5, 800 μmol·mol^{-1} CO_2 treated for 5 days; 400-day10, 400 μmol·mol^{-1} CO_2 treated for 10 days; 800-day10, 800 μmol·mol^{-1} CO_2 treated for 10 days; 400-day15, 400 μmol·mol^{-1} CO_2 treated for 15 days; 800-day15, 800 μmol·mol^{-1} CO_2 treated for 15 days.

* $P < 0.05$.

Table S3. Mantel tests between each PLFA or ^{13}C-PLFA and environmental factors at the end of labeling under 400 and 800 μmol·mol^{-1} CO_2 treatments (*p*).

	**10-Me-18:0**	**^{13}C-10-Me-18:0**	**18:1ω9c**	**^{13}C-18:1ω9c**	**18:2ω6,9**	**^{13}C-18:2ω6,9**	**20:0**	**^{13}C-20:0**
CO_2	0.2069	0.0273*	0.0279*	0.0276*	0.0881	0.1169	1	0.0291*
TDW[1]	0.2954	0.0135*	0.0282*	0.0121*	0.0067**	0.0087**	0.7446	0.0101*
CO_2_TDW	0.1084	0.0191*	0.0119*	0.235	0.8713	0.9848	0.6154	0.0188*

[1] Abbreviations: TDW, total dry weight; CO_2_TDW, the TDW was set as a covariate.

[*] Indicates significant correlation at $P < 0.05$.

[**] Indicates significant correlation at $P < 0.01$.

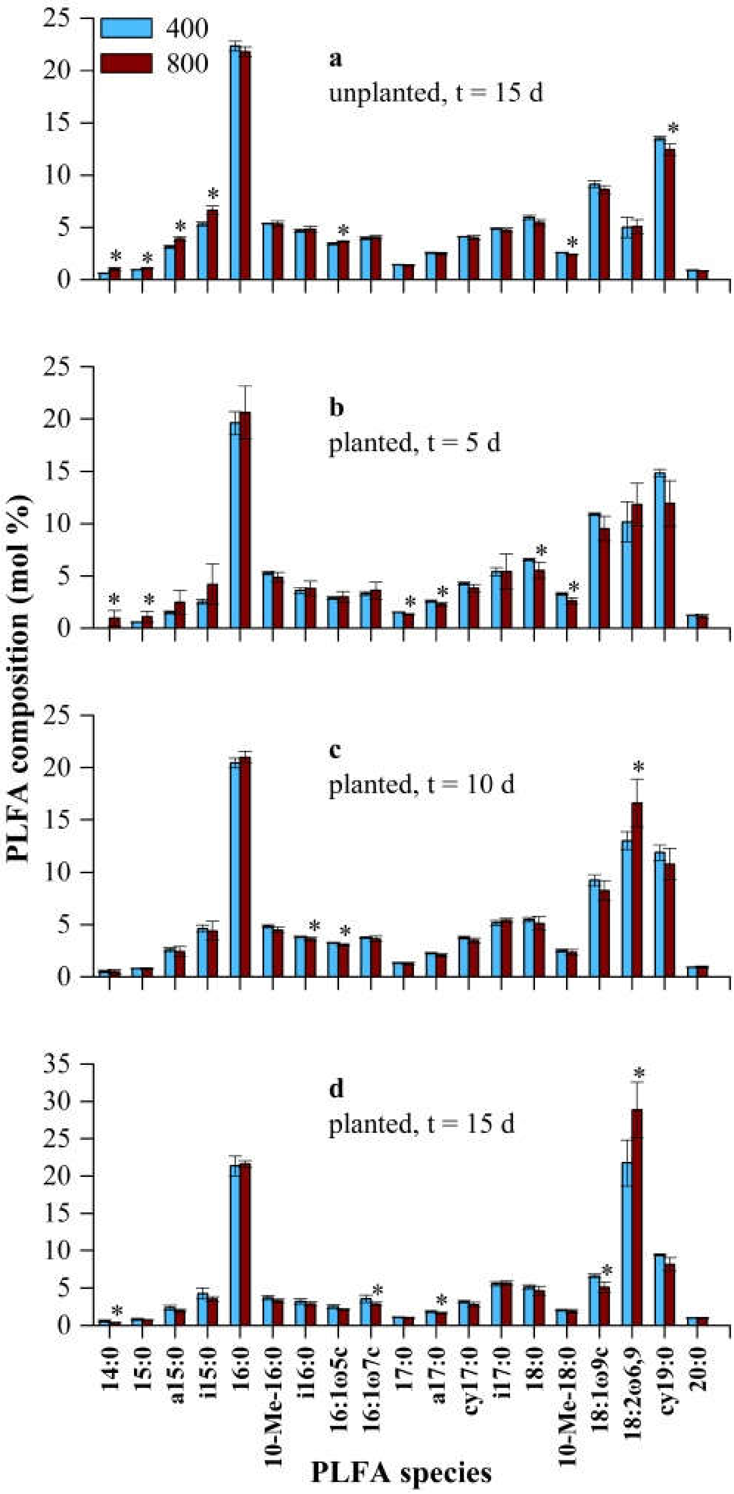

Figure S1. Differences in PLFA composition (mol%) between the 400 and 800 μmol·mol^{-1} CO_2 treatment groups in unplanted and planted soils on each sampling day. The labels in the figure represent significant PLFA biomarkers ($P < 0.05$). (**a**) PLFA composition (mol%) in unplanted soils sampled on labeling day 15; (**b**) PLFA composition (mol%) in planted soils sampled on labeling day 5; (**c**) PLFA composition (mol%) in planted soils sampled on labeling day 10; (**d**) PLFA composition (mol%) in planted soils sampled on labeling day 15. 400: 400 μmol·mol^{-1} CO_2 treatment; 800: 800 μmol·mol^{-1} CO_2 treatment. Bars represent the standard errors of the means (n=4). *: $P < 0.05$.

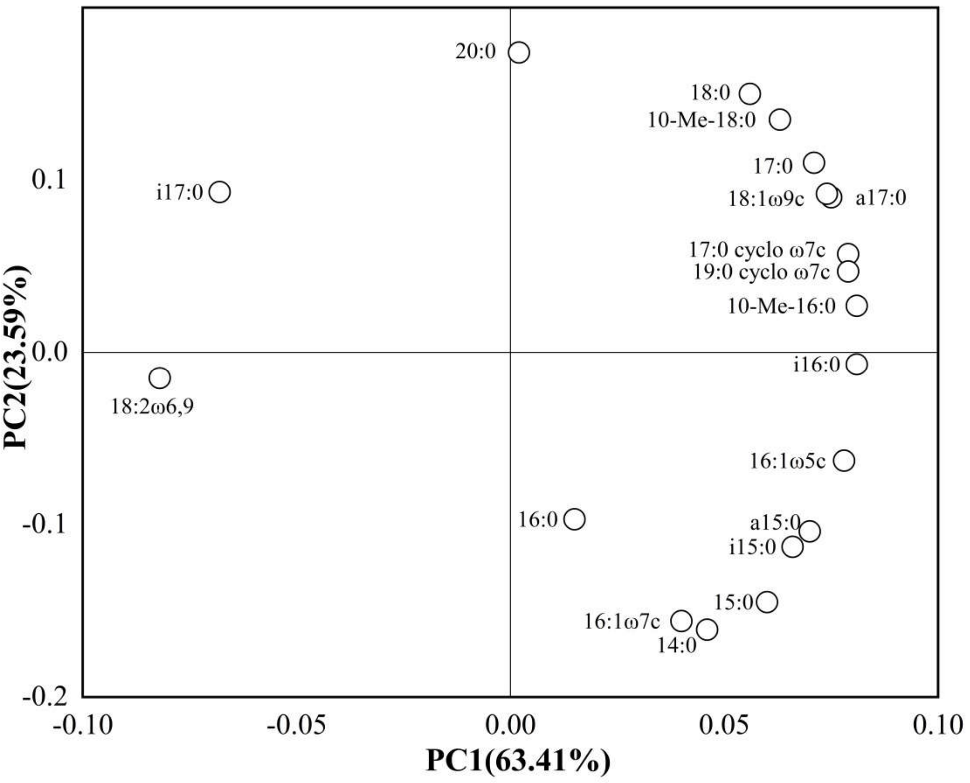

Figure S2. Loading scores of 19 PLFAs in the principal component analysis (PCA) at the end of the 15-day labeling experiment. Values in parentheses on the axis labels indicate the percentage variation accounted for by each axis.

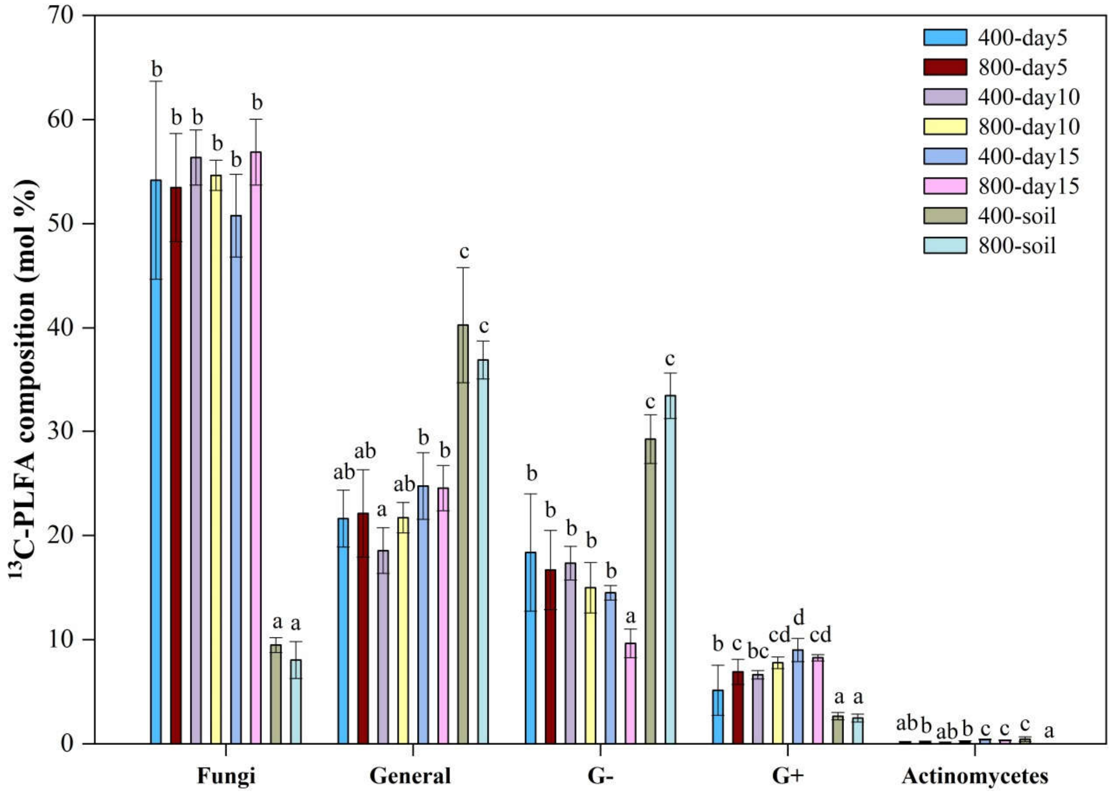

Figure S3. ^{13}C-PLFA compositions (mol%) of fungi, general PLFAs, G^-, G^+ and actinomycetes in planted and unplanted soils during the 15-day labeling experiment. Different lowercase letters shown in the figure represent significant variations ($P < 0.05$). Bars represent the standard errors of the means (n=4).

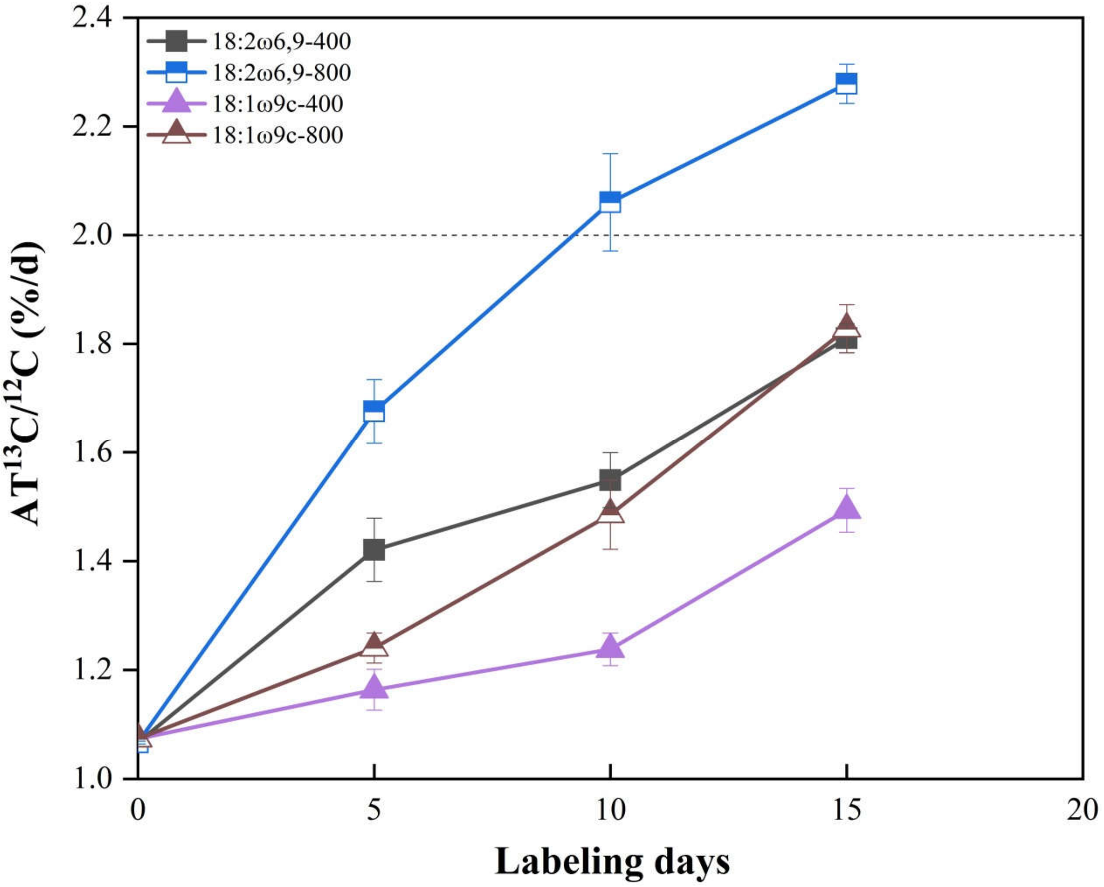

Figure S4. Changes in AT% $^{13}C/^{12}C$ values of 18:2ω6,9 and 18:1ω9c under various CO_2 treatments during the 15-day labeling experiment. 400: 400 μmol·mol^{-1} CO_2 treatment; 800: 800 μmol·mol^{-1} CO_2 treatment. Bars represent the standard errors of the means (n=4).
